# Adaptive methodology to determine hydrophobicity of nanomaterials *in situ*

**DOI:** 10.1371/journal.pone.0233844

**Published:** 2020-06-03

**Authors:** Lauren E. Crandon, Kylie M. Boenisch, Bryan J. Harper, Stacey L. Harper

**Affiliations:** 1 School of Chemical, Biological and Environmental Engineering, Oregon State University, Corvallis, Oregon, United States of America; 2 Department of Environmental and Molecular Toxicology, Oregon State University, Corvallis, Oregon, United States of America; 3 Oregon Nanoscience and Microtechnologies Institute, Corvallis, Oregon, United States of America; VIT University, INDIA

## Abstract

The hydrophobicity of nanoparticles (NPs) is a key property determining environmental fate, biological partitioning and toxicity. However, methods to characterize surface hydrophobicity are not uniformly applied to NPs and cannot quantify surface changes in complex environments. Existing methods designed to evaluate the hydrophobicity of bulk solids, chemicals, and proteins have significant limitations when applied to NPs. In this study, we modified and evaluated two methods to determine the hydrophobicity of NPs, hydrophobic interaction chromatography (HIC) and dye adsorption, and compared them to the standard octanol-water partitioning protocol for chemicals. Gold, copper oxide, silica, and amine-functionalized silica NPs were used to evaluate methods based on their applicability to NPs that agglomerate and have surface coatings. The octanol water partitioning and HIC methods both measured Au NPs as hydrophilic, but despite having a small size and stable suspension, NPs could not be fully recovered from the HIC column. For the dye adsorption method, hydrophobic (Rose Bengal) and hydrophilic (Nile Blue) dyes were adsorbed to the NP surface, and linear isotherm parameters were used as a metric for hydrophobicity. CuO was determined to be slightly hydrophilic, while SiO_2_ was hydrophilic and Ami-SiO_2_ was hydrophobic. The advantages and limitations of each method are discussed, and the dye adsorption method is recommended as the most suitable for application across broad classes of nanomaterials. The dye assay method was further used to measure changes in the surface hydrophobicity of TiO_2_ NPs after being suspended in natural water collected from the Alsea Rivers watershed in Oregon. TiO_2_ NPs adsorbed Rose Bengal when suspended in ultrapure water, but adsorbed Nile Blue after being incubated in natural water samples, demonstrating a shift from hydrophobic to hydrophilic properties on the outer surface. The dye adsorption method can be applied to characterize surface hydrophobicity of NPs and quantify environmental transformations, potentially improving environmental fate models.

## Introduction

Despite increasing commercial and industrial use of nanoparticles (NPs) in areas such as sunscreens, cosmetics, catalysts, pigments, and antimicrobials, little is known of their environmental impact [1]. Estimates predict a rise in global consumption of nanomaterials from approximately 308,322 metric tons in 2016 to 733,220 metric tons in 2021 [2]. At their end-of-life, NPs are released and encounter dynamic and complex environments that transform their surface. Currently there is not sufficient information to establish predictive structure-activity relationships for risk assessment, mostly due to lack of physicochemical characterization of nanomaterials in relevant conditions [3]. Distribution coefficients are widely used to model the environmental fate and bioavailability of chemicals, but parallel descriptors for nanomaterials have not been widely implemented [4]

One of the most powerful and useful descriptors of chemicals is relative hydrophobicity. Hydrophobicity is an important parameter in risk assessment that can be used to predict movement through soil, transport in aqueous environments, bioavailability to organisms and partitioning in physiological systems [5]. Hydrophobicity is also thought to be a key parameter for the prediction of environmental behavior and biological interactions of nanomaterials [6,7]. As with chemicals, hydrophilic NPs are more likely to remain in the water column and potentially have increased mobility, whereas hydrophobic particles are more likely to attach to sediment organic matter. When NPs enter the environment, they encounter environmental constituents such as natural organic matter (NOM), ions and polysaccharides, which adsorb to the NP surface and form a dynamic corona, altering the NP surface properties and influencing fate, transformations, and uptake [8]. The surface hydrophobicity of NPs likely influences the composition of the corona and affinity for the surrounding environmental surfaces [9,10].

This concept is further applied to describe the interaction of NPs with organisms. Hydrophobic NPs have shown to interact with the lipid bilayer of organisms, and evidence suggests increased uptake relative to hydrophilic NPs [11]. After uptake, NP surface hydrophobicity has been shown to directly affect toxicity, circulation time, and bioaccumulation [12,13]. Additionally, hydrophobicity dictates interaction of the NP surface with biological components such as proteins and biomolecules which adsorb to the surface, further altering biological interaction [14].

Nanoparticle-specific considerations add to the complexity of measurement and interpretation of hydrophobicity metrics. NPs can be comprised of various sizes and surface functionalization, both of which affect partitioning behavior and interactions. Hou *et al*. found that the size of Au NPs affected how quickly NPs distributed to a solid-supported lipid membrane, while surface functionality and solution chemistry determined the “apparent” steady state concentrations [15]. Agglomeration can also affect fate and complicate measurements by causing settling of NPs over time and preventing true partitioning behavior [16]. NPs are often functionalized with various surface coatings which can also alter surface hydrophobicity. Previous studies have found that changes in hydrophobicity of the particle surface can alter cell interactions and consequent uptake [17]. Another study found that the attachment of Ag NPs to hydrophobic collector surfaces was directly proportional to hydrophobicity of coatings (citrate, PVP, and GA) [18]. Therefore, a useful measure of hydrophobicity is needed that accurately represents complex and dynamic NP behavior.

Conventional methods to quantify the hydrophobicity of chemicals and solid surfaces rely on partitioning or other equilibrium dependent measurements. However, NPs do not reach thermodynamic equilibrium, so kinetically controlled parameters are more relevant and appropriate [19]. One approach is to obtain values for attachment efficiency (α) of NPs which can be directly applied to model the deposition of NPs to a collector surface as using the Smoluchowski equation. This method is well established for modeling the coagulation of particles and has been shown to successfully quantify the attachment of certain NPs to surfaces in complex environments [20–24]. However, α must be experimentally determined for each pair of surfaces and is dependent on media properties such as ionic strength, pH, and concentration of organic matter. There is a need to quantify the inherent surface hydrophobicity of NPs to predict attachment to surfaces using computational fate models in a manner parallel to forecasting the partitioning of chemicals into environmental compartments. To address this, Valsesia et al. developed a method to quantify attachment efficiency for NPs and standard engineered collector surfaces [25]. This method is promising, but has so far been applied only to idealized, spherical NPs in controlled conditions.

Existing methods for characterizing the hydrophobicity of substances have been applied to NPs and met with varying degrees of success. Examples of these methods include contact angle, which is typically used to evaluate the hydrophobicity of solid surfaces, octanol-water partitioning, which is commonly used for chemicals, and hydrophobic interaction chromatography, which provides a relative measure of the hydrophobicity of proteins. In some cases, these methods have been modified for NP specific behavior.

Contact angle measures the wettability of a solid surface by a probe liquid, typically using the sessile drop technique, and measuring the angle at the solid-liquid-vapor interface. To apply this technique to nanomaterials, a NP suspension is first filtered and pressed into a flat disk before applying the probe liquid. This method was performed across a series of rare earth oxide NPs and all were found to be hydrophobic, with water contact angles between 100° and 115° [26]. A similar method was applied to fullerenes, fullerols and coated Ag NPs at the liquid, liquid, solid interface and all were found to be hydrophilic, which was inconsistent with other measurements [27]. Arnaudov *et al*. used a gel trapping technique to eliminate the need to press NPs into a flat disk, but this method requires advanced techniques such as atomic force microscopy and does not consider effects of agglomeration or functionalization [28]. A major limitation of using contact angle for hydrophobicity is that it does not allow for experimental *in situ* measurements [26,27,29]. Additionally, it does not take into account NP size, shape, surface roughness, or heterogeneity [30].

If NP suspensions are modeled as homogenous solutions, the partitioning behavior between two immiscible liquid phases (octanol and water) could be used to evaluate hydrophobicity. The octanol-water partitioning coefficient (K_OW_) is commonly used for chemicals and is a powerful descriptor to model environmental fate and bioavailability for risk assessment [31]. Octanol is used as a surrogate for organic rich material, such as the sediment or lipid membrane. This measurement, when applied to dissolved chemicals, assumes that solutes move freely between two phases, and the equilibrium concentrations represent the “affinity” for each phase.

NP suspensions violate the basic assumptions of solubility and equilibrium associated with K_OW_, but many studies still apply this measure to evaluate the hydrophobicity of NPs [19]. The method has been most commonly applied to fullerenes, and although they are generally characterized as very hydrophobic, exact values are not consistent among studies and span orders of magnitude [27,32]. When applied to carbon nanotubes, measured K_OW_ values were not found to be predictive of bioaccumulation in earthworms or oligochaetes. The K_OW_ of nanomaterials has been reported to be inconsistent with organic compounds of a similar chemical structure, with aggregation, size and surface coatings all being cited as possible explanations [33].

Some studies have attempted to obtain a K_OW_ value for NPs while acknowledging the shortcomings or making efforts to adapt the results for particle-based systems. When applying the shake-flask method to measure K_OW_, some nanomaterials have been observed to partition at the interface between the aqueous and octanol phases. A two parameter distribution coefficient was proposed to analyze measurements in this scenario, where the mass ratio of NPs in the aqueous, organic, and interface are all taken into account [34]. The resulting measurements are system-dependent and a function of area of the interface, particle count, and time. Another proposed adaptation is to evaluate the K_OW_ of the surface functional groups alone, and assume that the core has a negligible effect on surface hydrophobicity [7]. This is likely most suitable for NPs with small cores and large, branched organic coatings. K_OW_ measurements of this nature may be useful to compare within a class of nanomaterials but have not been widely implemented to date.

An alternative method to evaluate hydrophobicity of NPs is hydrophobic interaction chromatography (HIC). HIC is typically used to separate proteins based on their relative hydrophobicity [35]. Proteins are eluted through a column by a stepwise decrease in salt concentration, where the most hydrophobic proteins are eluted at the lowest salt concentration. The stationary matrix of the column is typically comprised of agarose beads functionalized with alkyl chains of various lengths. While this method is potentially suitable for application with NPs due to the similar size range of proteins, it has only been applied to measure hydrophobicity of NPs in a limited number of studies. Polystyrene NPs were evaluated at a constant salt concentration and the elution volume required to completely remove particles from an alkyl-agarose column was used as a measure of hydrophobicity [36]. Another adaptation was to use multiple columns with varying alkyl chain lengths to compare hydrophobicity of various polymeric NPs [37]. HIC can potentially be used for particles with a wide range of hydrophobicity because the stationary phase can be selected by the user.

The adsorption of hydrophobic dyes to the particle surface is another method that is potentially well suited to NPs. This method has been applied to fluorescently labeled polystyrene NPs, latex particles with various functional groups, and solid lipid NPs using Rose Bengal, an organic dye, as a hydrophobic probe [17,38,39]. Some difficulties identified with this method were interference from surfactants, time intensive range-finding for suitable NP concentrations, and difficulty separating NPs from suspension for absorbance analysis. Rose Bengal provides robust measurements for hydrophobic NPs but provides limited information about hydrophilic surfaces. Use of A hydrophilic dye, Nile Blue, was demonstrated as a means to provide resolution to measurements of hydrophilic particles [27]. The use of both hydrophobic and hydrophilic dyes is promising to provide measurements with good resolution to compare NPs with a wide range of compositions.

In this study, we evaluated HIC and dye adsorption (Rose Bengal and Nile Blue) to evaluate the hydrophobicity of NPs and compared them to traditional K_OW_ methods. The results were evaluated based on the ability of each method to overcome major challenges associated with NPs: agglomeration, surface functionalization, and environmental transformations. Uncoated gold (Au) NPs were selected for their small size and ability to be easily quantified by absorption spectroscopy. Uncoated copper oxide (CuO) NPs were used for their known propensity to agglomerate in solution. Silica (SiO_2_) NPs with and without amine surface functionalization were chosen to observe changes in hydrophobicity due to surface coatings. We surveyed, modified and evaluated methods based on potential widespread applicability to NPs, with the ultimate goal of obtaining rapid, useful *in situ* measurements for future environmental fate models.

## Methods

### Nanoparticle preparation

Stock suspensions (1000 mg/L) of 14 nm Au (U.S. Research Nanomaterials, Inc. Houston, TX), CuO (<50 nm, Sigma Aldrich), 80 nm SiO_2_ and aminated SiO_2_ (NanoComposix, San Diego, CA) NPs were prepared in 20 mL ultrapure water (MQ, Milli-Q 18.2 Ω resistivity, Merck Millipore, Burlington, MA) and sonicated with a cup horn style sonicator equipped with a circulating water bath to maintain temperature (Vibracell VCX 750, Sonics & Materials, Inc., Newtown, CT) at 40% amplitude for 2 minutes (40.1 W, 4812 J). Stocks were further diluted in ultrapure water.

### Nanoparticle characterization

The hydrodynamic diameters (HDD) of CuO, Au, SiO_2_, and Ami-SiO_2_ NPs were evaluated in ultrapure water immediately following dispersion and sonication. A volume of 1.5 mL was placed in a disposable cuvette for measurement. Zeta potential measurements were performed in 0.5x phosphate buffered saline (PBS) to provide sufficient ionic strength to carry an electrical charge. HDD and ZP measurements were both performed using a Malvern Zetasizer (Nano ZS, Malvern Instruments, Worcestershire, UK) and the detailed parameters are described in S1 Table of S1 Data.

### Octanol-water partitioning

The OECD shake flask method [40] was applied to obtain a K_OW_ value for Au NPs. A volume of 4 mL each of ultrapure water and 1-octanol were equilibrated for 24 hours with 0.2 mg Au NPs. The liquid phases were allowed to separate for 4 hours, after which samples were collected from each phase and Au NPs were quantified using a SpectraMax M2 spectrophotometer (Molecular Devices, Sunnyvale, CA, USA) at λ = 530 nm. Absorbance was converted to concentration using a standard curve prepared in ultrapure water or 1-octanol. The same method was used to evaluate CuO NPs except the absorbance was evaluated at 640 nm.

### Hydrophobic interaction chromatography

HiTrap Octyl FF prepacked HIC columns were purchased from GE Life Sciences (Piscataway, NJ). The bed volume was 1 mL and the stationary phase consisted of a sepharose support matrix of 90 μm beads functionalized with hydrophobic octyl ligands, which were selected to parallel the octanol reference phase of the K_OW_ method. The column was loaded with 2 mL of a 10 mg/L Au NP suspension at a flow rate of 1 mL/min. A lower concentration was used (10mg/L) relative to the K_OW_ method to limit agglomeration, which would block flow through the pore space. After loading the column, a syringe pump (Model No. NE-1010, New Era Pump Systems, Inc. Farmingdale, NY, USA) was used to flow 20 mL of 0.5x PBS through the column at 1 mL/min and the eluent was collected in 1 mL fractions. To remove Au NPs retained in the column during PBS elution, a surfactant (0.1% Triton X-100, laboratory grade, Sigma-Aldrich, St. Louis, MO), was pumped through the column at 1 mL/min for another 20 minutes, and the eluent was again collected in 1 mL fractions every minute. The collected samples from both elution phases were placed in a 96-well plate and the absorbance at 530 nm was evaluated using UV-vis spectroscopy to determine Au concentration.

### Dye adsorption

**Fig 1:**
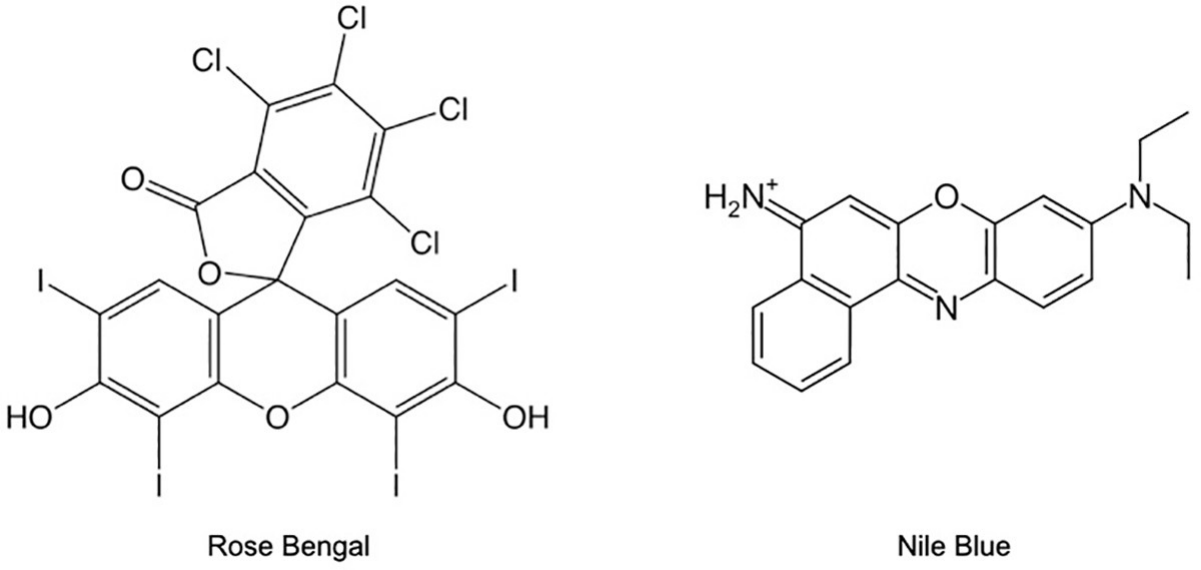
Structures of the hydrophobic (Rose Bengal) and hydrophilic (Nile Blue) probes.

The relative adsorption of a hydrophobic probe (Rose Bengal, 85% ACROS Organics, New Jersey, USA) and a hydrophilic probe (Nile Blue A, ACROS Organics, New Jersey, USA) to the NP surface was used as a measure of hydrophobicity (Fig 1). Dye concentrations (0.5–30 μM) were prepared in ultrapure water. Equal volumes of dye and NP stock were combined in 1.5 mL microcentrifuge tubes for each concentration and incubated in a tube rotator for 90 minutes. Controls were prepared by adding dye to ultrapure water to account for any observed loss of dye due to adsorption to the vials. To evaluate potential degradation of dye by reactive oxygen species (ROS) from the NP surface, controls were prepared with various concentrations of H_2_O_2_. Each sample was prepared in triplicate. Following incubation, NPs were removed from solution by centrifugation for 30 minutes at 14000 rpm. The remaining concentration of dye in the supernatant was analyzed using UV-Vis spectroscopy at λ = 543 nm for Rose Bengal and λ = 620 nm for Nile Blue. This method was performed with final concentrations of 500 mg/L SiO_2_ and Ami-SiO_2_ and 250 mg/L CuO. The dye concentrations were selected based on the minimum and maximum detectable absorbance by the UV-Vis spectrometer. NP concentrations were maximized to observe a significant change in absorbance while minimizing the effects of agglomeration. A lower CuO concentration was used to minimize agglomeration.

The amount of dye adsorbed to the NP surface (q_e_) was calculated using Eq 1:

$$q_{e}=(C_{0}-C_{e})\frac{V}{m}$$ (1)

where C_0_ is the initial dye concentration, C_e_ is the concentration of dye reaming in the supernatant after centrifugation, V is the volume (L) and m is the mass of NPs (g). The adsorption of dyes was fit to a linear (Eq 2), Langmuir (Eq 3) and Freundlich (Eq 4) type isotherm models. Each model was fit by minimizing the sum of squared errors in SigmaPlot (Systat Software, San Jose, CA, USA) to determine the linear adsorption constant, k_lin_ (L/g), the adsorption capacity, q_max_ (μmol dye/gNP), the Langmuir adsorption capacity, K_f_ (L/umol) and the adsorption intensity, 1/n for the Freundlich model.

$$q_{e}=k_{lin}C_{e}$$ (2)

$$q_{e}=\frac{q_{max}K_{L}C_{e}}{\left(1+K_{L}C_{e}\right)}$$ (3)

$$q_{e}=K_{f}{qC}_{e}^{1/n}$$ (4)

### Environmental transformations

To simulate the environmental release of NPs and evaluate the effect on dye adsorption, natural water samples were collected from various sources along the Alsea River Watershed in Oregon (S5 Fig of S1 Data). Conductivity and pH were measured at the time of collection. The samples were later filtered using a 0.44 mm Whatman GF/F glass fiber filter to remove particulate matter prior to performing alkalinity and hardness tests. Suspension of TiO_2_ NPs (250 mg/L) were prepared in each of the water samples and allowed to incubate for 24 hours. NPs were then removed by centrifugation and resuspended in ultrapure water, after which the previously described method for dye adsorption was performed.

### Statistical analysis

SigmaPlot version 13.0 (Systat Software, San Jose, CA, USA) was used to perform all statistical analyses. All experiments were performed in triplicate. Area under the curve (AUC) measurements for HIC analysis were performed in SigmaPlot using built in graphical integration functions.

## Results and discussion

### Nanoparticle characterization

**Fig 2:**
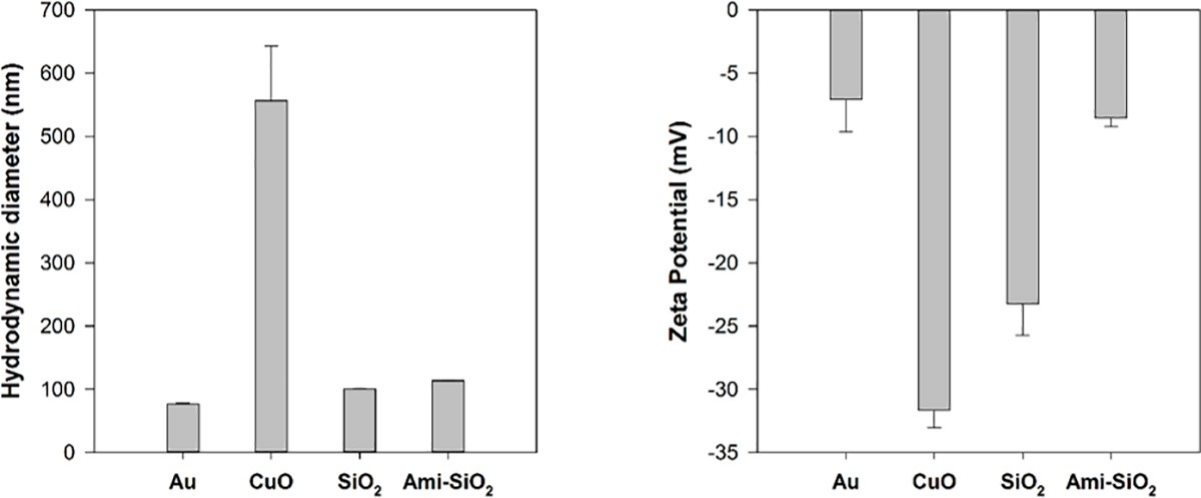
Hydrodynamic diameter of Au, CuO, SiO_2_, and Ami-SiO_2_ evaluated in ultrapure water and zeta potential evaluated in 0.5x PBS.

Au, SiO_2_, and Ami-SiO_2_ NPs were all stable in suspension and had an average HDD of 76.8 ± 1.5 nm, 100.3 ± 0.3 nm, and 113.5 ± 0.5 nm in ultrapure water, respectively, which is similar to their primary particle size (Fig 2A). CuO NPs showed a high degree of agglomeration, with an average HDD of 556 ± 87 nm in ultrapure water compared to a primary particle size of <50 nm. Agglomeration is expected to be more significant in complex media such as the 0.5x PBS buffer used for zeta potential measurements [41]. The ZP of the CuO NPs was -31.7 mV, indicating that despite high agglomeration, the suspension was stable. The zeta potential shows that all particles had a negative surface charge in 0.5x PBS (Fig 2B). The surface charge of Ami-SiO_2_ was neutralized relative to SiO_2_. The isoelectric point of Ami-SiO_2_ is pH 7.5 compared to 2.5 for SiO_2_, so below this pH the surface would be expected to be positively charged. The pH of the PBS solution was approximately 7.8.

### Octanol water partitioning (K_OW_)

Au and CuO NPs were selected to evaluate octanol-water partitioning because the particles can be easily quantified using UV-Vis spectroscopy. Au particles partitioned to the aqueous phase and remained suspended (S1 Fig of S1 Data). No visible Au NPs were observed at the octanol-water interface. A log K_OW_ of approximately -2.1± 0.6 was calculated, suggesting Au NPs are hydrophilic.

The log K_OW_ of Au NPs measured in this study was compared to published values for Au, and it was found that the reported hydrophobicity of elemental gold is not consistent among studies. Native gold flakes were found to be hydrophobic and floated in water [42]. However, Smith *et al*. performed a comprehensive review of studies that characterized the contact angle of Au and found opposing conclusions about its hydrophobic or hydrophilic nature. Performing Auger electron spectroscopy before and after each measurement revealed that organic impurities were causing hydrophobic measurements, and clean gold surfaces were determined to be inherently hydrophilic [43]. The modeled value of log K_OW_ for elemental Au was found to be slightly hydrophobic (log K_OW_ Au = 0.03), while ionic forms were hydrophobic (Log K_OW_ AuCl_3_ = 0.16) or hydrophilic (log K_OW_ AuCl = -0.46) depending on the valency [44].

CuO NPs were visually observed to aggregate at the liquid-liquid interface and settled to the bottom of the vial over time (S2 Fig of S1 Data). Additionally, CuO in the octanol phase could not be accurately quantified because they did not disperse in octanol (S3 Fig of S1 Data). The measured log K_OW_ was -0.34 ± 0.39. Modeled log K_OW_ values of the bulk and dissolved phases were estimated to be -1.10 for CuO, 0.52 for CuCl_2_, and 0.16 for elemental copper [44]. This is consistent with experimentally determined contact angle measurements of Cu films, which showed a change from slightly hydrophobic to slightly hydrophilic as the surface oxidized to CuO [45]. CuO is known to exhibit some dissolution of Cu^+2^ in aqueous systems which may also affect the hydrophobicity at the surface [46].

Despite some limited success in this study and others, the octanol-water partitioning method cannot be widely applied across classes of NPs or systems and should be limited to comparisons among classes of NPs. The overall conclusion of hydrophobic or hydrophilic was in agreement with other measures of hydrophobicity in the literature, so this method may be useful as a preliminary qualitative observation, but measured values do not provide sufficient resolution or consistency for use in fate models. NP suspensions do not reach a thermodynamic equilibrium between liquid phases, and values for K_OW_ are dependent on NP concentration, time, and size of the vial which affects the area of the liquid-liquid interface. This was particularly evident for CuO NPs, which exhibit significant agglomeration and settling.

### Hydrophobic interaction chromatography

**Fig 3:**
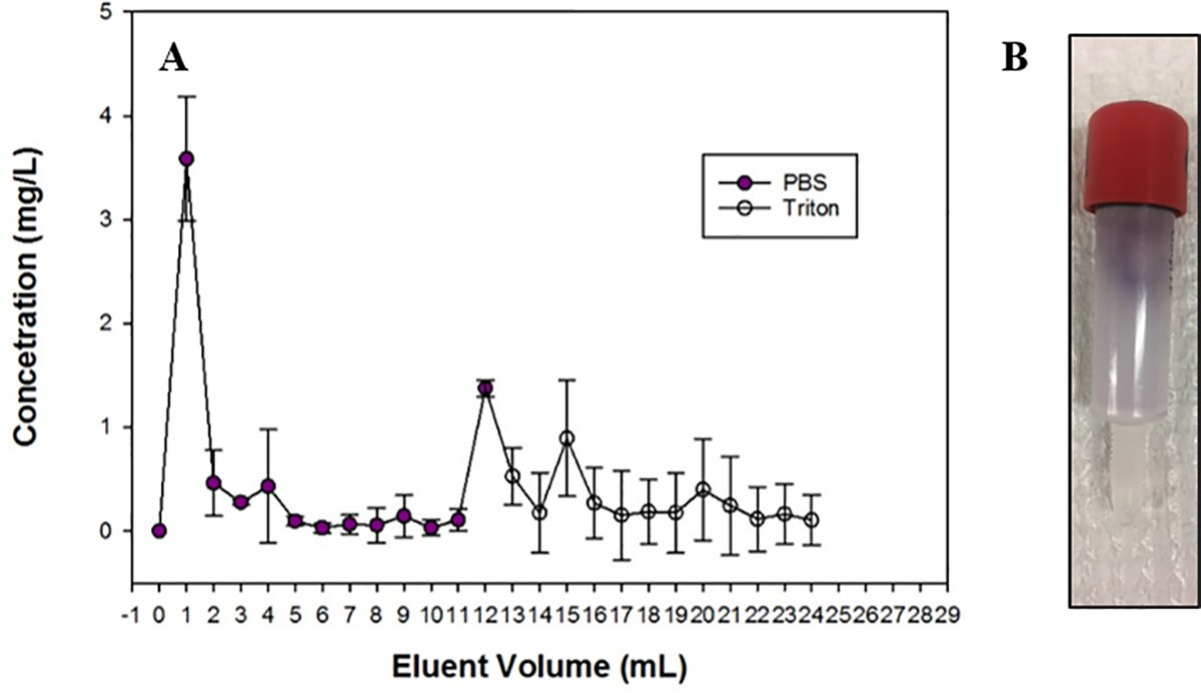
A) Concentration of Au in the eluent with PBS and Triton mobile phases as a function of total eluent volume. B) Gold NPs visibly retained in column after flushing with 20 mL surfactant and 20 mL 20% ethanol.

The HIC method was adapted to measure hydrophobicity of NPs by interaction with hydrophobic octyl ligands. Salt concentration was not varied, and instead mass of particles retained in the column was compared to mass of particles eluted by an aqueous phase (0.5x PBS). Concentration of Au plotted as a function of cumulative eluent volume (Fig 3) shows that a high concentration of Au NPs was initially flushed out in the first column volume, which was likely residual from the loading step. Small concentrations of Au NPs were measured in the eluent throughout the PBS phase. Some Au NPs were initially eluted with the surfactant but those particles only represented a small portion of Au retained in the column. The concentration of Au retained in the column was difficult to determine by UV-Vis analysis because of interference from the surfactant bubbles, so higher variance was therefore observed in this elution phase relative to the PBS phase (Fig 3). Degassing the mobile phase under vacuum may minimize this problem for future studies.

For analysis, the area under the curve (AUC) of concentration vs. volume was evaluated to determine total Au mass in the PBS and surfactant eluents. The ratio was used as a measure of hydrophobicity according to Eq 5:

$${LogK}_{OW,HIC}≅\frac{{AUC}_{surfactant}}{{AUC}_{PBS}}$$ (5)

Log K_OW, HIC_ for Au NPs was calculated to be -0.447 ± 0.006, indicating they are hydrophilic.

Although the HIC method is rapid, directly comparable to log K_OW_, and potentially more appropriate for nanomaterial behavior, several factors limit its widespread use. Au NPs were not fully recovered from the column, and their size likely restricted travel through the pore space. Particles were visually observed to be retained at the top the column, even after flushing with 20 mL surfactant and further attempts to regenerate the column with 20 mL 20% ethanol (Fig 3B). Previous studies have also observed higher rates of NP column retention than predicted by classical filtration theory, which models interaction of a single particle with a collector surface, with deposition governed by DLVO forces (electrostatic and Van der Waals) [47]. Chowdhury et al. observed an increase in retention of TiO_2_ NPs in sand columns with increased agglomeration, despite unfavorable electrostatic interactions [48]. This was attributed to straining, which refers to physical retention in the pore structure, and has been reported to occur when the ratio of NP diameter to bead diameter is greater than approximately 0.002 [49]. In this study, the ratio of the primary particle size (14 nm) to bead diameter (90 μm) is 0.00016, but using the aggregate size the ratio is 0.0009. Straining likely plays a role in the observed column retention, and would certainly be more severe for particles with more significant agglomeration, such as metal oxides, but may not be the only explanation. Alternative reasons for high retention include charge heterogeneity of the stationary phase and the presence of organic impurities [47].

Theoretically, the columns can ideally be regenerated, but we were unable to completely remove particles after loading. In addition, it was difficult to accurately quantify NPs in the eluent. UV-Vis spectroscopy is simple and widely available, but many NPs may have absorbance interference with plates or cuvettes, and concentrations in this case were too low for sensitive quantification. Absorbance was not a reliable measure in the surfactant eluent due to interference from bubbles forming in solution. Other methods may be more suitable, such as inductively coupled plasma mass spectrometry (ICP) for metal particles, nanoparticle tracking analysis (NTA) for stable and spherical particles, or other analytical methods for carbonaceous NPs but these are more costly and less accessible.

We were unable to perform HIC due to difficulties in recovering and quantifying NPs concentration in the eluent. Columns with larger pores may improve the usefulness of this method. Salt concentrations can be increased or decreased to alter hydrophobic interactions within the stationary phase. The column stationary phase can be selected to be functionalized with alkyl ligands of various chain lengths, and a lower degree of ligand substitution could also decrease the retention of particles in the column. However, this would be less comparable to K_OW_ and these parameters would need to be optimized for specific nanomaterials.

### Dye adsorption

The dye adsorption method was successfully performed for agglomerated and functionalized particles. Adsorption isotherms were fit to linear, Langmuir and Freundlich models (Table 1). Linear models can generally be applied at low adsorbate concentrations [50]. The Langmuir model assumes monolayer adsorption on a relatively regular surface [51]. The Freundlich model describes adsorption on a heterogeneous surface. In general, the data were best represented by the linear and Freundlich models (Table 1). The linear adsorption constant, k_lin_, was used as a measure of affinity for each probe to the NP surface. The relative affinity of RB and NB to the surface was evaluated and then Eq 6 was used as a unitless measure of hydrophobicity, shown here as a hydrophobicity ratio (HR).

$$LogHR_{RB/NB}=Log\frac{k_{lin,RB}}{k_{lin,NB}}$$ (6)

Previous studies that used RB adsorption to measure hydrophobicity of NPs varied the NP concentration and held the dye concentration constant, plotting fraction of dye adsorbed (known as the partition quotient) as a function of surface area [27]. We adapted these methods and instead plotted adsorption isotherms by varying the dye concentration and using a constant NP concentration. This eliminates the need to calculate NP surface area, which is difficult to measure *in situ* and can contribute to uncertainty in measurements. Additionally, this reduces NP waste and simplifies preliminary range finding for optimizing NP concentration. Adsorption isotherms are well established for suspended particles, and do not require assumptions of spherical geometry or monodispersity to estimate surface area.

**Table 1:** Summary of isotherm parameters.

Linear	CuO	SiO_2_	Ami-SiO_2_
k_lin_,_RB_	0.090 ± 0.127	0.254 ± 0.044	1.99 ± 0.49
R^2^	0.02	0.65	0.67
k_lin,NB_	2.90 ± 0.07	13.23 ± 39.70	0.05 ± 0.02
R^2^	0.99	0.86	0.40
**Langmuir**			
q_max, RB_	3.57 ± 1.13	----	44.13 ± 53.01
K_L, RB_	1.23 ± 1.96	----	0.067 ± 0.10
R^2^	0.11		0.68
q_max, NB_	876.86 ± 4944.49	44.81 ± 5.86	2.18 ± 5.77
K_L, NB_	0.004 ± 0.024	0.77 ± 0.20	0.03 ± 0.10
R^2^	0.89	0.93	0.41
**Freundlich**			
1/n, _RB_	0.53 ± 0.48	---	1.14 ± 0.27
K_F, RB_	1.53 ± 0.50	---	2.16 ± 1.48
R^2^	0.02	---	0.67
1/n, _NB_	0.77 ± 0.03	0.31 ± 0.16	1.49 ± 0.03
K_F, NB_	5.61 ± 0.38	17.06 ± 4.17	0.016 ± 0.001
R^2^	0.98	0.97	0.59
**Log HR**	-1.51	-1.72	1.60

**Fig 4:**
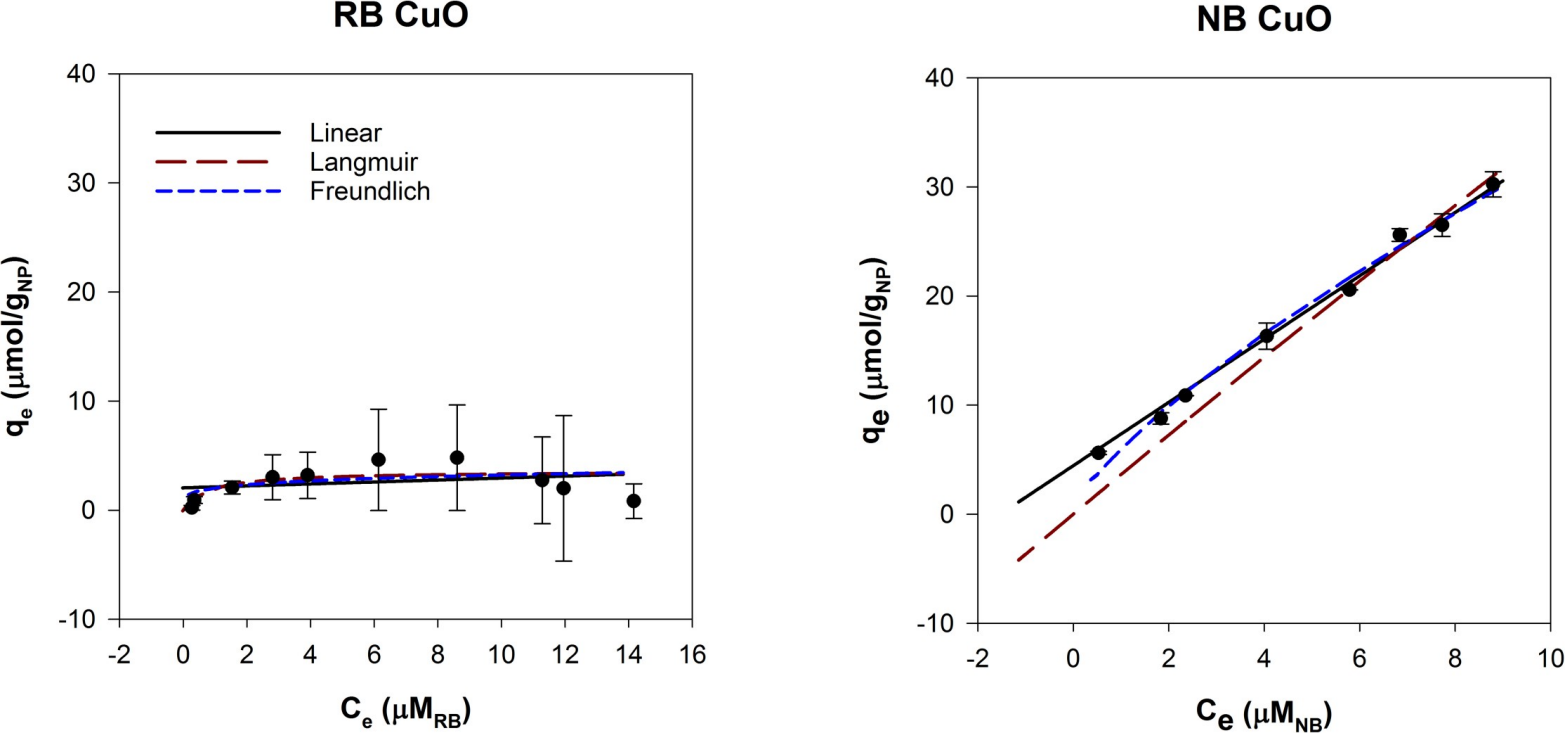
Adsorption isotherms for 250 mg/L CuO with Rose Bengal and Nile Blue modeled with linear, Langmuir and Freundlich adsorption models.

For CuO NPs, both dyes adsorbed to the surface, but NB had higher adsorption than RB (Fig 4). CuO NPs had a high degree of agglomeration (Fig 2), which can affect the available surface for adsorption. However, this effect is likely similar for both dyes, and is essentially normalized when Eq 6 is applied. This suggests that the dye adsorption method can be used for particles that agglomerate in solution.

**Fig 5:**
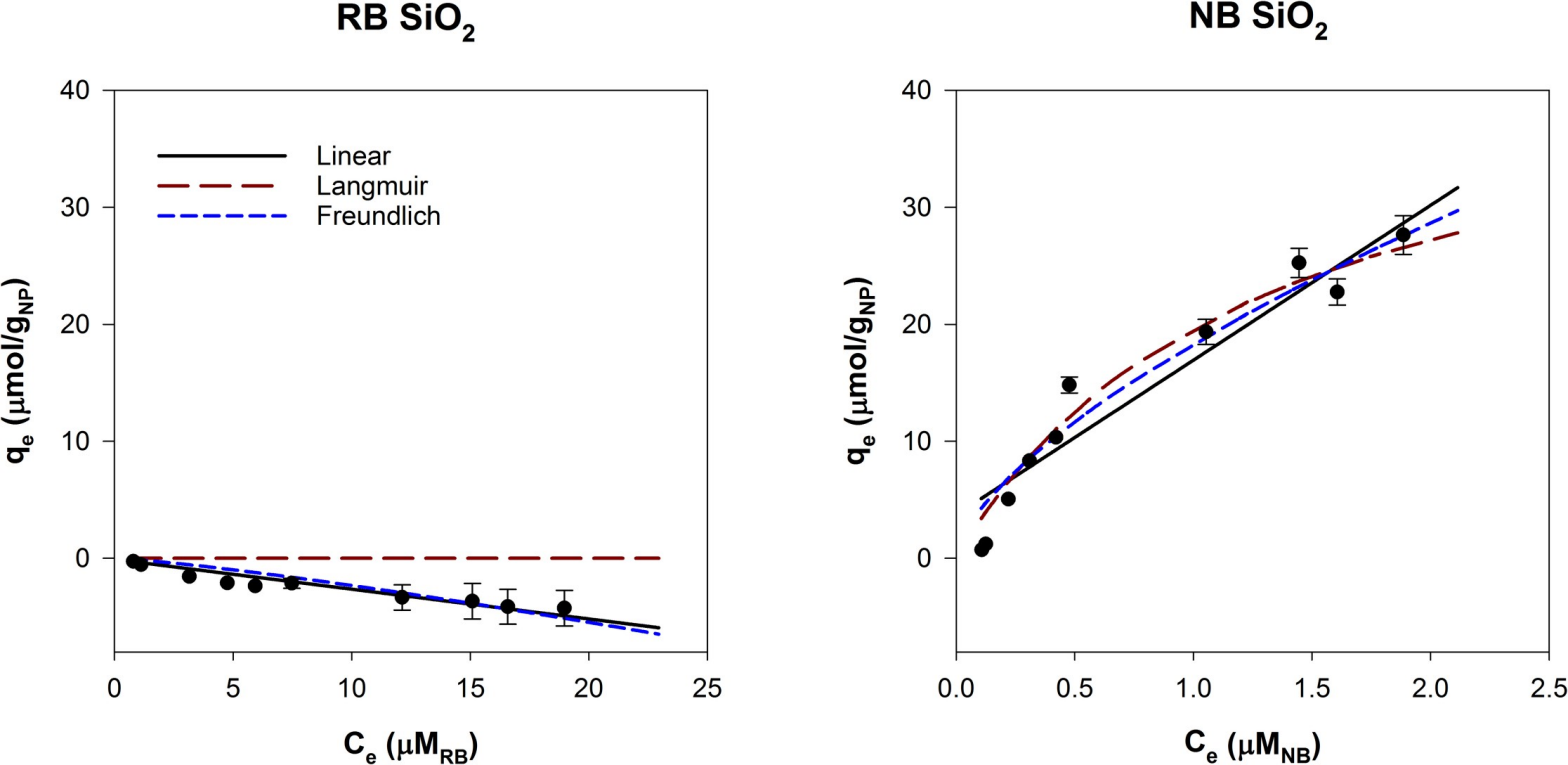
Adsorption isotherms for 500 mg/L SiO_2_ NPs with Rose Bengal and Nile Blue, modeled with linear, Langmuir and Freundlich adsorption models.

SiO_2_ particles with and without amine functional groups were compared using the dye adsorption method (Fig 5). Both have low solubility at pH 2–8 and remained stable in suspension. Bare silica was predicted to be hydrophilic (log K_OW_ is -0.66), which is consistent with our results [44]. Nile Blue adsorbed to the SiO_2_ surface while negligible RB was adsorbed.

**Fig 6:**
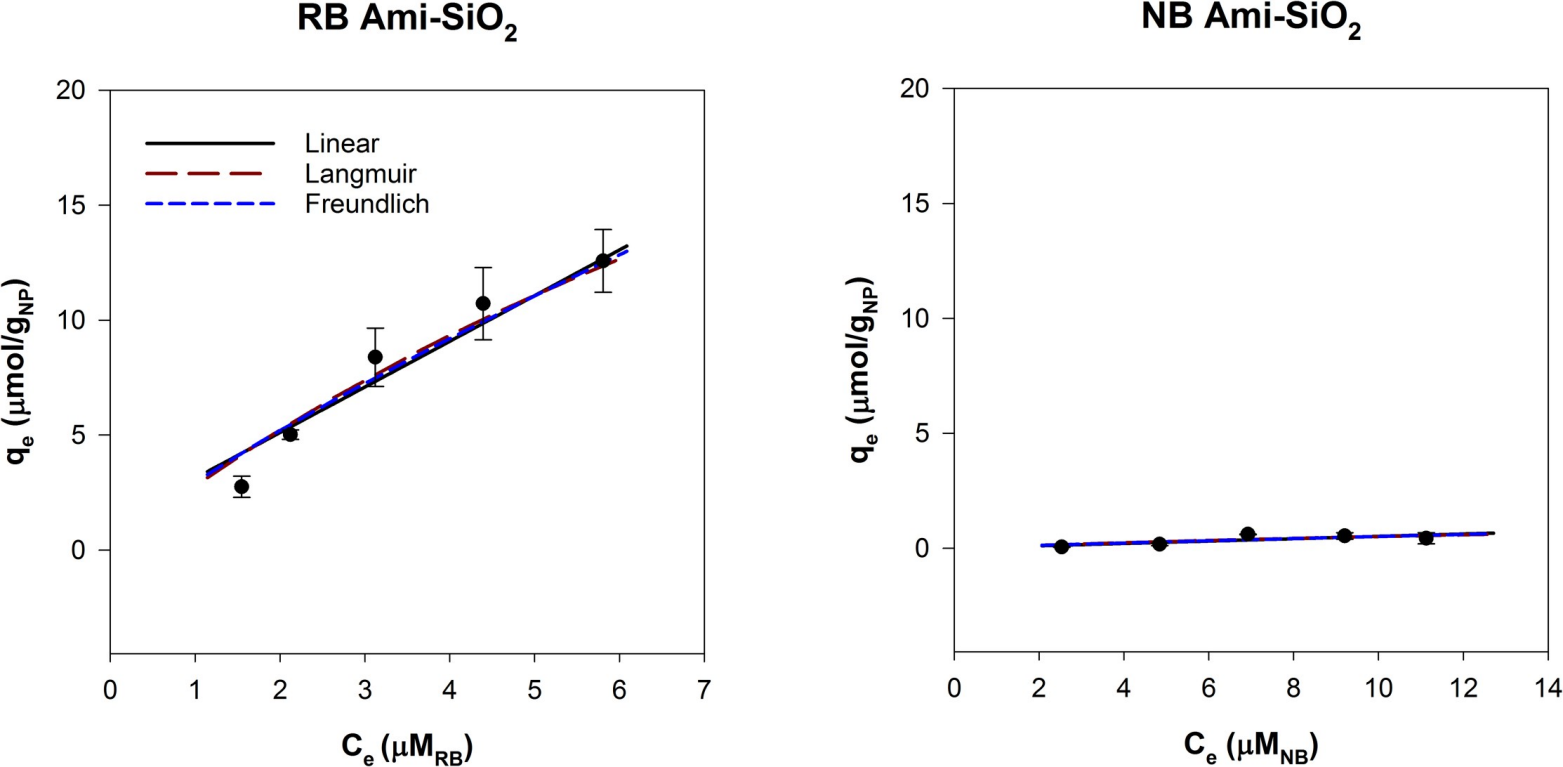
Adsorption isotherms for 500 mg/L Ami-SiO_2_ NPs with Rose Bengal and Nile Blue, modeled with linear, Langmuir and Freundlich adsorption models.

Amine groups at the surface drastically altered the hydrophobic interactions of the SiO_2_ core. Ami-SiO_2_ showed the opposite trend of SiO_2_ NPs and had high adsorption of RB and minimal NB adsorption (Fig 6). The k_lin_ for RB was 1.99 ± 0.49 and log (k_lin,RB_/k_lin,NB_) was 1.60. The observed hydrophobic nature is consistent with the log K_OW_ for amine groups, which become more hydrophobic with increasing alkyl chain lengths [44]. The results indicate that the dye adsorption method is well suited to evaluate NPs with covalently bound functional groups. This method can measure the change in surface hydrophobicity of NPs due to functionalization and potentially incorporate factors such as size and surface coverage.

A potential shortcoming of the dye adsorption method is that it is difficult to distinguish between adsorption due to hydrophobic interaction or due to electrostatic interaction. The probes used here are oppositely charged: RB is anionic and NB is cationic. The Ami-SiO_2_ NPs had a positively charged surface and therefore likely experienced electrostatic interaction with RB. Amine functionalized mesoporous hollow silica shells were previously found to completely remove another anionic dye, Congo Red, from solution and this was attributed to the oppositely charged surface [52]. However, Congo Red is also hydrophobic (log K_OW_ = 3.57) which could have contributed to the high rate of adsorption. In the current study, CuO suspensions were negatively charged but adsorbed some anionic Rose Bengal, indicating that despite repulsive electrostatic charges, hydrophobic interactions led to adsorption.

### Environmental transformations

Environmental transformations caused differences in hydrophobicity measurements and can be measured. The transformations were observed even after NPs were removed from the natural water and re-suspended in MQ. Fresh water samples collected along the Alsea River Watershed were used to evaluate potential environmental transformations of NPs. Samples were analyzed (S2 Table of S1 Data) and varied mostly in conductivity, with Waldport Bay having the highest (53500 μS). TiO_2_ NPs were selected here because they are one of the mostly widely produced engineered NPs, and their use in personal care products makes them likely to be present in surface waters at detectable concentrations [53]. The HDD of TiO_2_ after 24 hours of incubation was 1223 nm and 2256 nm when measured in ultrapure and Waldport Bay water, respectively (S3 Table of S1 Data). The increase in HDD may have decreased the overall NP surface area available for dye adsorption.

The dye assay was performed in all water samples and the linear adsorption parameters are shown in S4 Table of S1 Data. When TiO_2_ NPs were suspended in natural fresh water, there was greater adsorption of NB in all water sources and less adsorption of RB relative to MQ (S6–S8 Figs of S1 Data). Significant differences (p≤0.05) between both k_lin,RB_ and k_lin,NB_ demonstrated a shift from the hydrophobic nature of TiO_2_ NPs observed by high adsorption of RB in ultrapure water. This change is likely due to ions and organic matter which interacted with the NP surface to alter its properties.

Prior to measuring dye adsorption, NPs suspended in natural waters were removed and resuspended in ultrapure water because preliminary studies revealed that high salt concentrations caused NB dye to precipitate out of solution. However, environmental transformations could still be observed even when the assay was performed in ultrapure water. This method is promising to provide a quantitative measure of changes at the NP surface.

### Comparison of methods

The major advantages and limitations of the three methods shown here are summarized in Table 2. Most notably, the dye adsorption assay does not require direct quantification of NPs. This is a major advantage because various methods are used to quantify nanomaterials depending on material composition and availability of instruments, and this can lead to inconsistent values among studies. For metal and metal oxide NPs that dissolve in solution, metal ions could interfere with measurements to quantify NPs, particularly in the octanol-water partitioning method. If dissolution is sufficient to alter the local ionic strength, adsorption processes occurring in both the HIC and dye adsorption methods could be affected [54]. For the dye adsorption method, an ionic control could be used to account for potential complexes that form between the probe dyes and metal ions.

**Table 2:** Comparison of methods to evaluate NP hydrophobicity.

Method	Advantages	Limitations
K_OW_	• Simple • Directly comparable to existing measurements and fate models	• Violates equilibrium assumptions • Must directly quantify NPs • Settling of NPs affects measurements • Measurement depends on particle count, area of interface, etc.
HIC	• Rapid • Minimal NP waste	• Movement limited through pore space • Must directly quantify NPs
Dye Adsorption	• Do not have to directly quantify NPs • Suitable for NPs that agglomerate • Does not require extensive range finding • Can be applied in natural systems	• Difficult to interpret if no adsorption • Charged probes contribute to adsorption • Octanol is not reference phase

The dye adsorption method, unlike the other methods, produced meaningful results despite high agglomeration of CuO NPs. This method allows for *in situ* measurements without the need for stabilizing agents and can potentially be applied to evaluate the surface hydrophobicity of NPs in more complex environments. Although the probes selected here may produce measurement artifacts due to opposite surface charges, relative differences between NPs can be observed and alternative probes may be explored in the future. We conclude that dye adsorption is a viable method to rapidly asses hydrophobicity of NPs with various surface chemistries, degrees of agglomeration, and environments.

## Conclusion

Surface hydrophobicity is a key parameter influencing environmental fate and biopartitioning, and a standard metric is needed to compare across NPs. A number of disparate protocols are applied in the literature, depending on the chemistry and stability of the NP suspension. This study surveyed and modified candidate methods to be used as a standard measurement of NP surface hydrophobicity. Measuring the adsorption of hydrophilic and hydrophobic dyes to the NP surface is proposed as the most viable method to broadly compare the hydrophobicity of NPs. When compared to alternative methods currently being employed, we conclude that the dye adsorption assay is the best in terms of ability to overcome difficulties associated with agglomeration, functionalization, and quantification of nanomaterials. Future work should evaluate this method for evaluating NPs with non-spherical morphology and additional chemistries and sizes. In this study, changes in dye adsorption could be observed after suspension of NP in natural waters, providing a possible path forward to quantify complex environmental transformations. The octanol water partitioning method was determined to only be suitable for select particles that are small, stable and easily quantified, limiting its widespread use. HIC is theoretically suitable for future use with NPs but is problematic for agglomerated NPs and would require thorough optimization of a reference column. The dye adsorption method is rapid and can be implemented to assess the hydrophobicity of broad classes of NPs. It should be further validated with the ultimate objective of application in future predictive fate models.

## Supporting information

S1 Data (DOCX)
