# Supplementary material for: Adaptive methodology to determine hydrophobicity of nanomaterials in situ
Source: PLoS One. 2020 Jun 3;15(6):e0233844. doi: 10.1371/journal.pone.0233844 (PMC7269256; doi:10.1371/journal.pone.0233844)
Supplement: S1 Data — (DOCX) [file pone.0233844.s001.docx]

**Adaptive methodology to determine hydrophobicity of nanomaterials in situ**

Lauren E. Crandon^1^, Kylie Boenisch ^1^, Bryan J. Harper^2^ and Stacey L. Harper ^1,2,3^

^1^School of Chemical, Biological and Environmental Engineering, Oregon State University, Corvallis, Oregon, United States, ^2^Environmental and Molecular Toxicology, Oregon State University, Corvallis, Oregon, United States, ^3^Oregon Nanoscience and Microtechnologies Institute, Eugene, Oregon, United States

*Corresponding author. Email address: [stacey.harper@oregonstate.edu](mailto:stacey.harper@oregonstate.edu)

Supporting Information

| Shape | spherical |
| --- | --- |
| Model used to compute zeta potential | Henry’s Equation (Smoluchowski approximation) |
| Applied voltage | 148 V |
| Replicate measurements | 3 |
| Equilibration time | 120 s |
| Concentration NPs | 10 mg/L (Au) 50 mg/L (CuO) 100 mg/L (SiO_2_ and Ami-SiO_2_) |
| 0.5x PBS | |
| pH | 7.8 ± 0.2 |
| Temperature | 25 °C |
| Ionic strength | 83 mM |
| Viscosity | 0.8508 cP |
| Macromolecules/NOM present | none |

***Table S1*** *Standardized information for determining the zeta potential in 0.5x PBS*

*
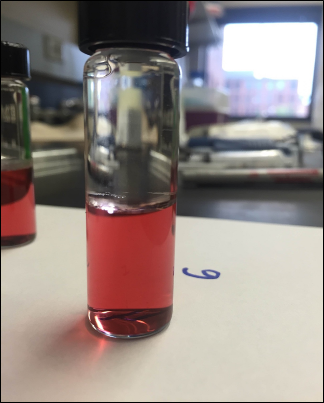
*

***Fig S1*** *The shake flask method for octanol water partitioning performed using Au NPs. Particles were visually observed to partition to the aqueous phase.*


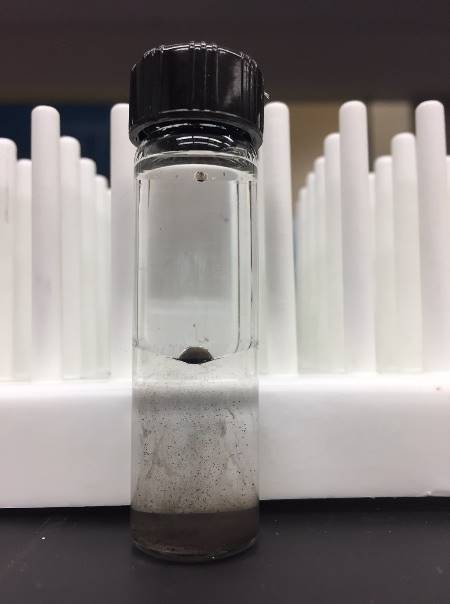

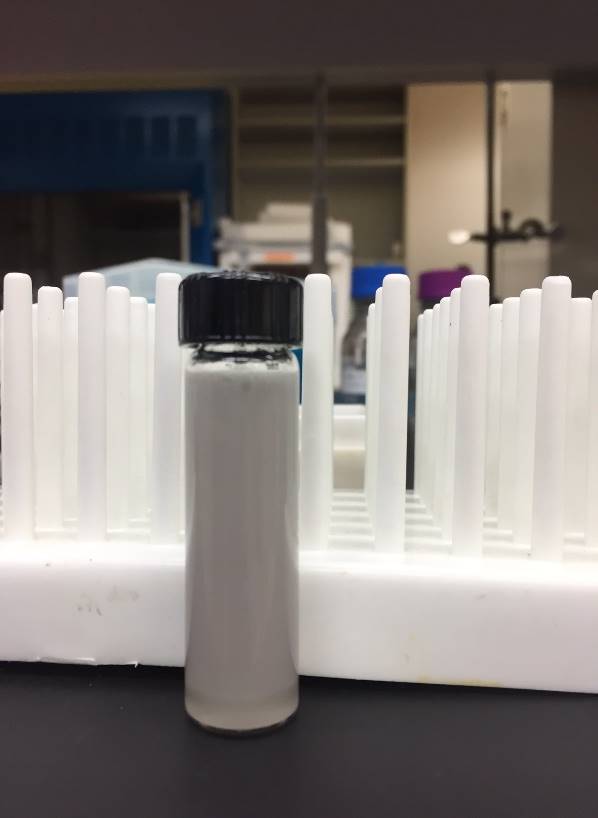

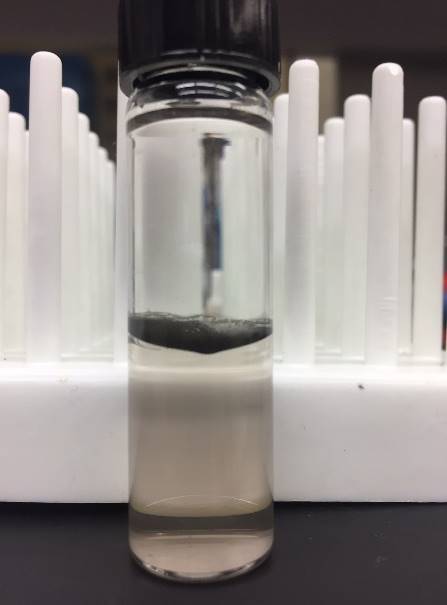

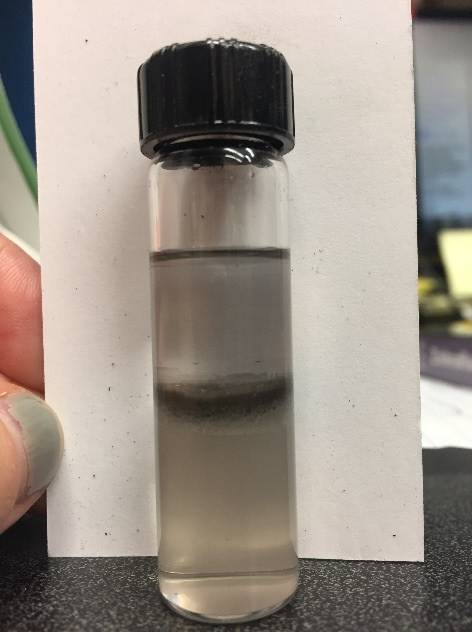


CuO NPs added to octanol and water after 24 hrs equilibration

CuO NPs mixed with octanol and water for 4 hrs

Liquid phases allowed to separate for 3 hrs

1 mL sample taken from each liquid phase

***Fig S2*** *The shake flask octanol-water partitioning method performed with CuO NPs. NPs are visually observed to sit at the octanol-water interface*


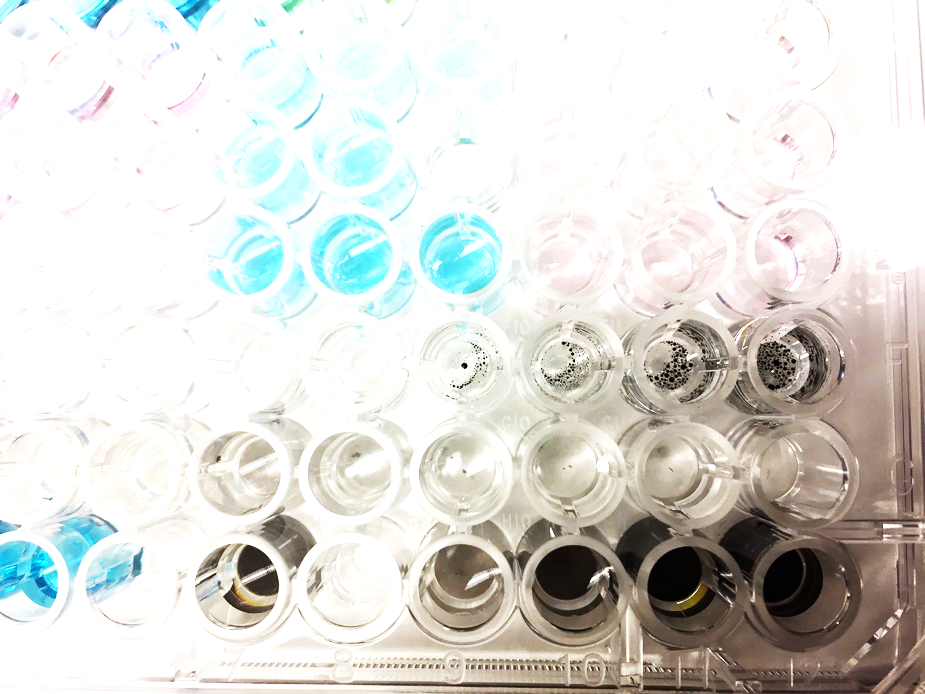


octanol

water

**Fig S3** CuO NPs suspended in octanol and water. A standard curve could not be performed to quantify CuO concentration in octanol because NPs could not be uniformly dispersed

The dye adsorption assay was performed using H_2_O_2_ as a positive control. The H_2_O_2_ concentrations used were higher than have been observed to be generated by CuO NPs (Denluck, Wu, Crandon, Harper, & Harper, 2018).


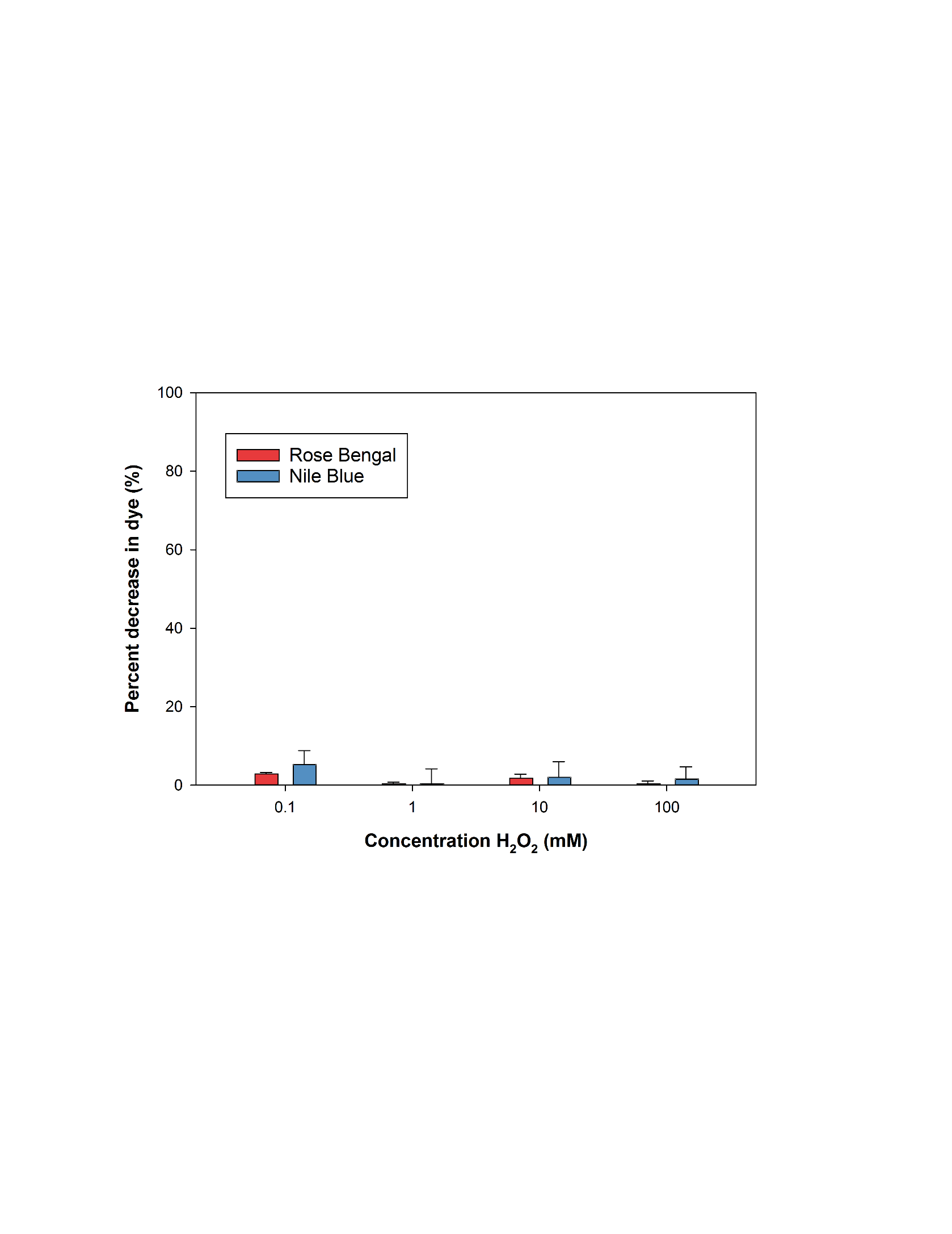


**Fig S4** Percent decrease in dye concentration as a function of hydrogen peroxide concentration.

*Environmental Transformations*

Water samples were collected on August 13, 2018. Conductivity and pH were measured onsite and all other water quality parameters were measured one day after water collection. Dye adsorption experiments were performed within three days of water collection.


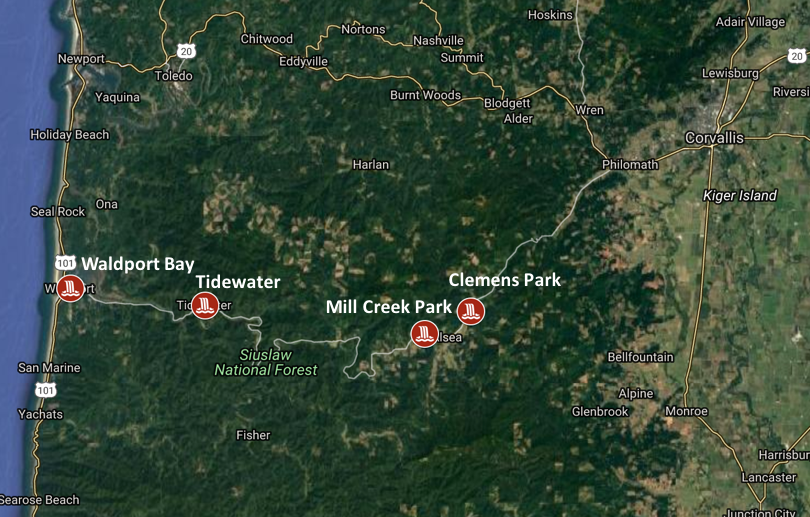


***Fig. S5.*** *Water collection locations along the Alsea River Watershed.*

***Table S2:*** *Water Quality Parameters*

| Location | GPS Coordinates | pH | Conductivity (µS) | Alkalinity (mg/L CaCO_3_) | Hardness (mg CaCO_3_/L) |
| --- | --- | --- | --- | --- | --- |
| Clemens Park | 44°24'34'' N 123°34'4'' W | 7.84 | 110 | 29 | 58 |
| Mill Creek Park | 44°23'5'' N 123°37'24'' W | 7.75 | 90 | 24 | 36 |
| Tidewater | 44°23'5'' N 123°37'26'' W | 7.31 | 13000 | 24 | 1030 |
| Waldport Bay | 44°25'54'' N 123°3'32'' W | 7.69 | 53500 | 38 | 3960 |

TiO_2_ NPs (10 mg/L) were suspended in each of the water samples and incubated for 24 hours. Hydrodynamic diameter and zeta potential measurements were subsequently performed using a Malvern Zetasizer (Nano ZS, Malvern Instruments, Worcestershire, UK).

***Table S3****: HDD and ZP for 10 mg/L TiO_2_*

| Location | HDD (nm) | Zeta Potential (mV) |
| --- | --- | --- |
| MQ H_2_O | 1223 | -8.58 |
| Clemens Park | 3354 | -15.3 |
| Mill Creek Park | 413 | -7.4 |
| Tidewater | 2398 | -15.8 |
| Waldport Bay | 2256 | -7 |

Significant differences in adsorbance (p≤0.05) were observed when the hydrophobicity dye assay was performed in the light vs. the dark with Milli-Q water and TiO_2_ P-25 NPs. This difference is likely due to the photocatalytic activity of TiO_2_ P-25 NPs. Thus, all experiments were run in the dark to negate the potential impact of this property on natural water results.


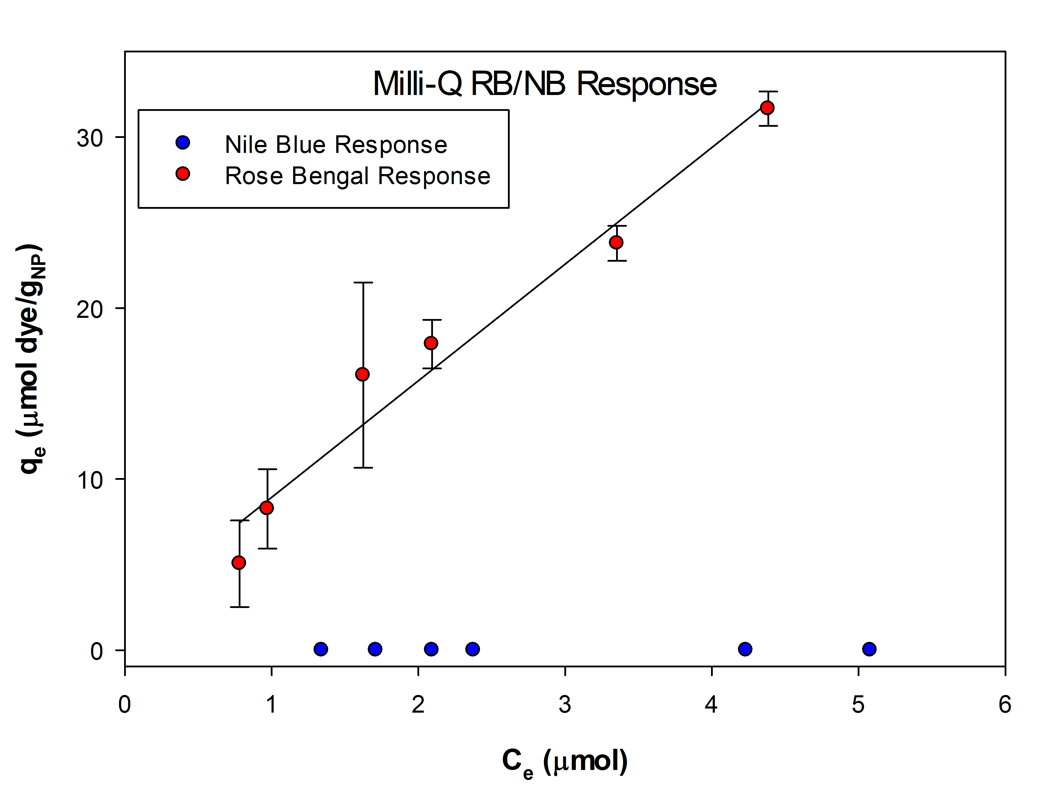


**Fig S6** 250 mg/L TiO2 P-25 NPs in Milli-Q Water with Nile Blue and Rose Bengal (0-15 μM). Nile Blue did not adsorb to the surface, but Rose Bengal was fit to a linear isotherm model (R^2^=0.96).


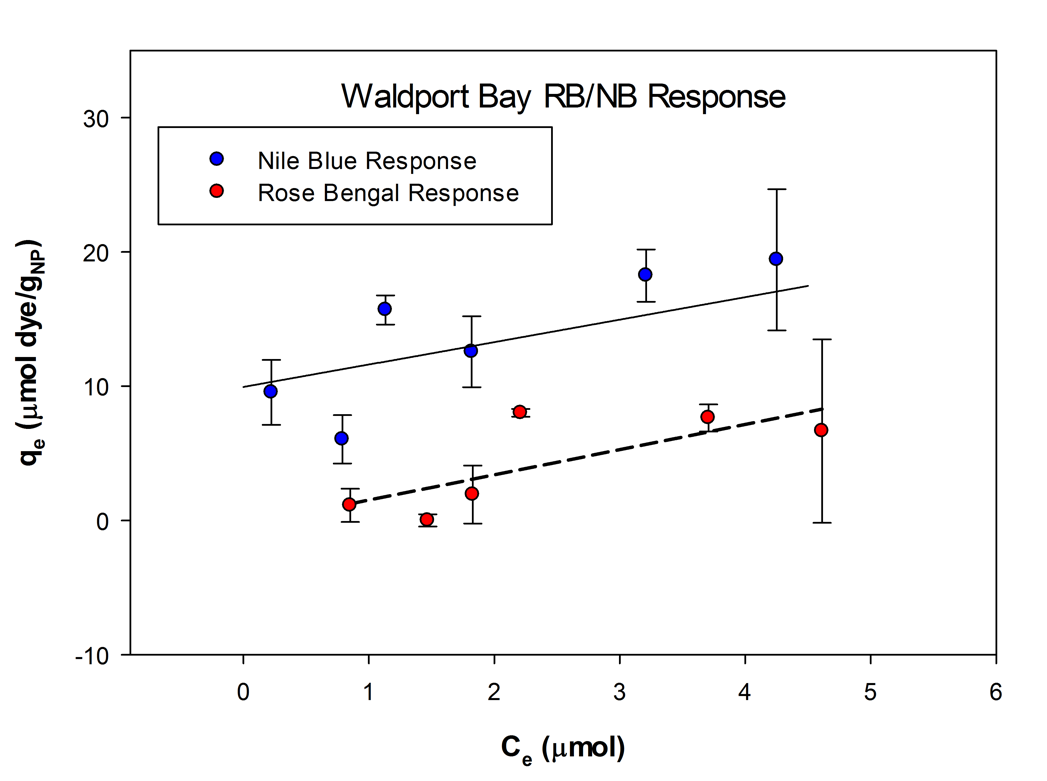


**Fig S7** 250 mg/L TiO2 P-25 NPs in water from Waldport Bay with Nile Blue and Rose Bengal (0-15 μM). Linear isotherm models were used to measure adsorption of Nile Blue (blue) and Rose Bengal (red) dyes (R^2^=0.7 and R^2^=0.55, respectively).

Figures S6 and S7 compare responses of TiO_2_ NPs in Milli-Q water and after incubation in Waldport Bay water, respectively. Significant differences (p≤0.05) between both k_lin,RB_ and k_lin,NB_ for each treatment of NPs was observed. This indicates environmental transformations caused differences in hydrophobicity measurements and can be measured. The transformations were observed even after NPs were removed from the natural water and resuspended in MQ.

This method was performed in all water samples and the linear adsorption parameters are shown in Table S4.

***Table S4:*** *Linear adsorption parameters for adsorption of RB and NB to 250 mg/L TiO_2_ in natural waters*

| Water Collection Location | k_lin,RB_ | k_lin,NB_ | HR  (k_lin,RB_/ k_lin,NB_) | Log HR |
| --- | --- | --- | --- | --- |
| Clemens | 0.001 | 0.1075 | 9.212x10^-3^ | -2.035 |
| Mill Creek | 0.001 | 0.001 | 1 | 0 |
| Tidewater | 1.1978 | 0.3949 | 3.033 | 0.482 |
| Waldport | 1.8360 | 2.8178 | 0.651 | -0.186 |
| MQ | 6.9059 | 0.001 | 6906 | 3.839 |


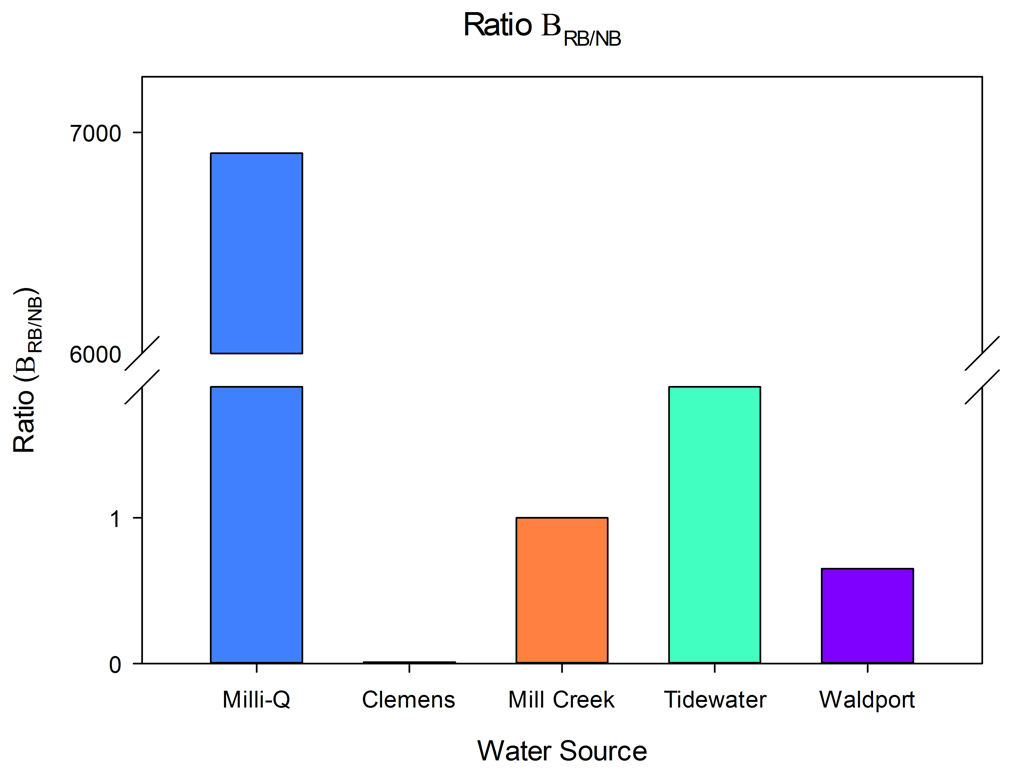


***Fig S8*** *Hydrophobicity ratio based on linear adsorption isotherms for 250 mg/L TiO_2_ in various natural waters. A higher ratio indicates a more hydrophilic particle surface.*

Significant transformation (p≤.05) in hydrophobicity of TiO_2_ from Milli-Q water laboratory tests found after incubation in all four natural water samples respectively (Fig S8.). A substantial hydrophilic response at Waldport Bay represents a drastic shift from the clear hydrophobic response of TiO_2_ seen in Milli-Q water.
